# The genome sequence of the nine-spined stickleback, *Pungitius pungitius *(Linnaeus, 1758)

**DOI:** 10.12688/wellcomeopenres.20354.1

**Published:** 2023-12-05

**Authors:** Bernd Hänfling, Alan Smith, Wellcome Sanger Institute Tree of Life programme, Wellcome Sanger Institute Scientific Operations: DNA Pipelines collective, Tree of Life Core Informatics collective, Darwin Tree of Life Consortium

**Affiliations:** 1 Institute for Biodiversity and Freshwater Conservation, University of Highlands and Islands, Inverness, Scotland, UK; 2 School of Natural Sciences, University of Hull, Hull, England, UK

**Keywords:** Pungitius pungitius, nine-spined stickleback, genome sequence, chromosomal, Gasterosteiformes

## Abstract

We present a genome assembly from an individual male *Pungitius pungitius* (the nine-spined stickleback; Chordata; Actinopteri; Gasterosteiformes; Gasterosteidae). The genome sequence is 480.4 megabases in span. Most of the assembly is scaffolded into 21 chromosomal pseudomolecules. The mitochondrial genome has also been assembled and is 16.57 kilobases in length.

## Species taxonomy

Eukaryota; Metazoa; Eumetazoa; Bilateria; Deuterostomia; Chordata; Craniata; Vertebrata; Gnathostomata; Teleostomi; Euteleostomi; Actinopterygii; Actinopteri; Neopterygii; Teleostei; Osteoglossocephalai; Clupeocephala; Euteleosteomorpha; Neoteleostei; Eurypterygia; Ctenosquamata; Acanthomorphata; Euacanthomorphacea; Percomorphaceae; Eupercaria; Perciformes; Cottioidei; Gasterosteales; Gasterosteidae; *Pungitius*; *Pungitius pungitius* (Linnaeus, 1758) (NCBI:txid134920).

## Background

The nine-spined stickleback ( *Pungitius pungitius*) is a small freshwater fish that belongs to the family Gasterosteidae and is widely distributed throughout the Northern Hemisphere with populations in North America, Europe, and Asia ( Kottelat & Freyhof, 2007; Page & Burr, 1991). The species’ conservation status was last assessed by the IUCN in 2012 and is classed as “least concern” due to its broad geographic distribution, large population sizes, large number of sub-populations and lack of major threats ( IUCN, 2022). The species is known for its tolerance to a wide range of temperatures, salinities, and water qualities and inhabits both coastal marine and freshwater environments including rivers, lakes, ponds, and estuaries ( Wootton, 1976; Wootton, 1984). *P. pungitius* primarily feeds on benthic and planktonic invertebrates, sharing a similar diet with three-spined stickleback, *Gasterosteus aculeatus* ( Hart, 2003; Hynes, 1950; Wootton, 1984). Where these two species occur sympatrically, *P. pungitius* displays a heightened preference for weeded areas, which potentially serves as a mechanism to avoid competition ( Copp *et al.*, 1998; Hart, 2003). The reproductive behaviour is characterised by paternal care where males construct and guard nests containing small clutches of eggs ( Maitland, 2004; Wootton, 1984).

The ability to colonise new habitats and adapt to changing environmental conditions has led to the establishment of several evolutionary lineages and ecotypes of nine-spined stickleback. The genetic structure of the species has been shaped by geographic isolation among several glacial refugia resulting in two allopatric phylogeographic lineages in Europe ( Teacher *et al.*, 2011) and by multiple independent colonisations of freshwater from marine habitats ( Bruneaux *et al.*, 2013; Mobley *et al.*, 2011; Shikano *et al.*, 2010). Adaptive evolution associated with these demographic processes has in many cases resulted in the repeated evolution of similar morphological and behavioural phenotypes across different populations ( Mobley *et al.*, 2011). Recent research indicates that this phenotypic convergence is largely underpinned by non-parallel genetic changes ( Wang *et al.*, 2020).

Due to its wide distribution, ecological versatility, and genetic diversity, the nine-spined stickleback has emerged as an ideal model organism for studying various aspects of evolution, behaviour and genetics ( Merilä, 2013; Peichel & Marques, 2017). Its suitability as a model system is further bolstered by its ease of captive breeding and the existence of nine congeneric species, compared to the three for *G. aculeatus*, which provides the opportunity for extensive comparative studies ( Merilä, 2013).

We present a chromosomally complete genome sequence for *P. pungitius*, based on one male specimen from Cottingham, UK, as part of the Darwin Tree of Life Project. This project is a collaborative effort to sequence all named eukaryotic species in the Atlantic Archipelago of Britain and Ireland. Together with a recently published high-quality genome assembly by ( Varadharajan *et al.*, 2019), the *P. pungitius* genome presented in this study will provide an important resource for comparative genomic and evolutionary analyses of fish and other vertebrates.

## Genome sequence report

**Figure 1.**
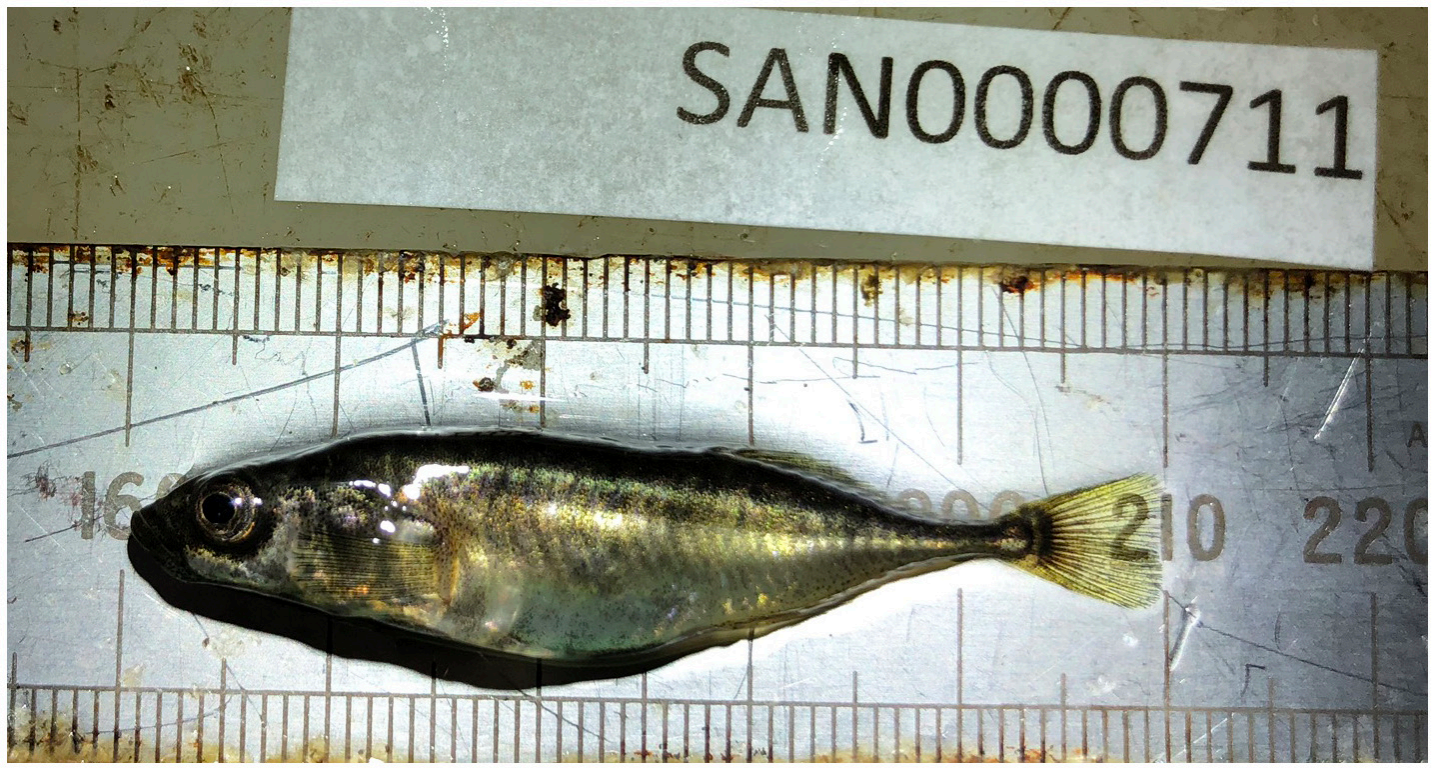
Photograph of the *Pungitius pungitius* (fPunPun2) specimen used for genome sequencing.

The genome was sequenced from one male *Pungitius pungitius* ( Figure 1) collected from Thwaite Lake, Cottingham, UK (53.78, –0.40). A total of 61-fold coverage in Pacific Biosciences single-molecule HiFi long reads was generated. Primary assembly contigs were scaffolded with chromosome conformation Hi-C data. Manual assembly curation corrected 44 missing joins or mis-joins and removed 3 haplotypic duplications, reducing the assembly length by 0.13% and the scaffold number by 6.42%, and increasing the scaffold N50 by 3.9%.

**Figure 2.**
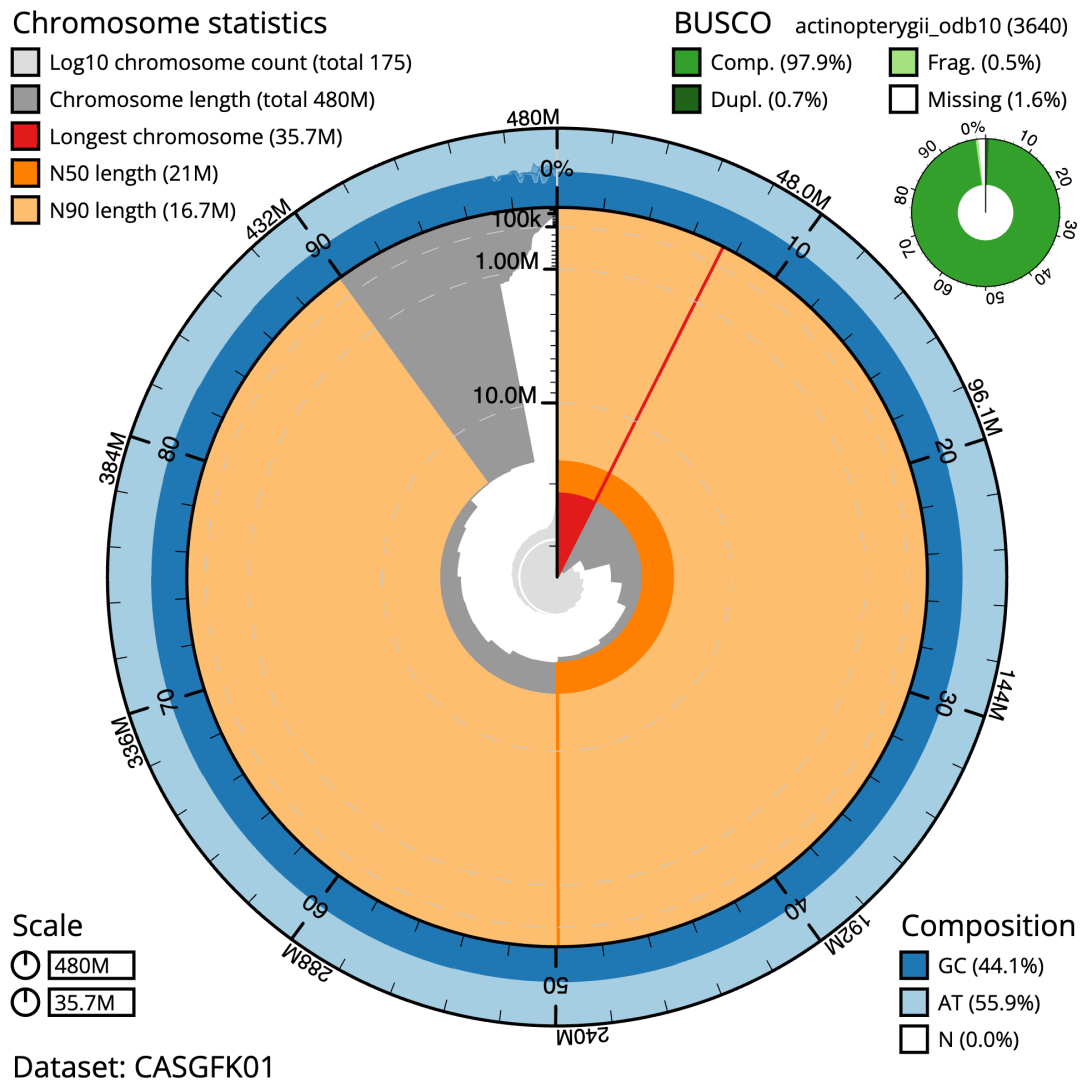
Genome assembly of *Pungitius pungitius*, fPunPun2.1: metrics. The BlobToolKit Snailplot shows N50 metrics and BUSCO gene completeness. The main plot is divided into 1,000 size-ordered bins around the circumference with each bin representing 0.1% of the 480,450,674 bp assembly. The distribution of scaffold lengths is shown in dark grey with the plot radius scaled to the longest scaffold present in the assembly (35,745,030 bp, shown in red). Orange and pale-orange arcs show the N50 and N90 scaffold lengths (21,005,440 and 16,732,017 bp), respectively. The pale grey spiral shows the cumulative scaffold count on a log scale with white scale lines showing successive orders of magnitude. The blue and pale-blue area around the outside of the plot shows the distribution of GC, AT and N percentages in the same bins as the inner plot. A summary of complete, fragmented, duplicated and missing BUSCO genes in the actinopterygii_odb10 set is shown in the top right. An interactive version of this figure is available at https://blobtoolkit.genomehubs.org/view/fPunPun2.1/dataset/CASGFK01/snail.

The final assembly has a total length of 480.4 Mb in 174 sequence scaffolds with a scaffold N50 of 21.0 Mb ( Table 1). Most (97%) of the assembly sequence was assigned to 21 chromosomal-level scaffolds. Chromosome-scale scaffolds confirmed by the Hi-C data are named in order of size ( Figure 2– Figure 5; Table 2). While not fully phased, the assembly deposited is of one haplotype. Contigs corresponding to the second haplotype have also been deposited. The mitochondrial genome was also assembled and can be found as a contig within the multifasta file of the genome submission.

**Table 1.** Genome data for *Pungitius pungitius*, fPunPun2.1.

Project accession data		
Assembly identifier	fPunPun2.1	
Species	*Pungitius pungitius*	
Specimen	fPunPun2	
NCBI taxonomy ID	134920	
BioProject	PRJEB59310	
BioSample ID	SAMEA11296545	
Isolate information	fPunPun2: male: muscle tissue (DNA sequencing, Hi-C data and RNA sequencing)	
Assembly metrics *		*Benchmark*
Consensus quality (QV)	55.9	*≥ 50*
*k*-mer completeness	99.99%	*≥ 95%*
BUSCO **	C:97.9%[S:97.1%,D:0.7%],F:0.5%, M:1.6%,n:3,640	*C ≥ 95%*
Percentage of assembly mapped to chromosomes	97.0%	*≥ 95%*
Sex chromosomes	Not identified	*localised homologous pairs*
Organelles	Mitochondrial genome assembled	*complete single alleles*
Raw data accessions		
PacificBiosciences SEQUEL II	ERR10812866	
Hi-C Illumina	ERR10818327	
PolyA RNA-Seq Illumina	ERR11242521	
Genome assembly		
Assembly accession	GCA_949316345.1	
*Accession of alternate haplotype*	GCA_949316245.1	
Span (Mb)	480.4	
Number of contigs	913	
Contig N50 length (Mb)	1.4	
Number of scaffolds	174	
Scaffold N50 length (Mb)	21.0	
Longest scaffold (Mb)	35.8	

* Assembly metric benchmarks are adapted from column VGP-2020 of “Table 1: Proposed standards and metrics for defining genome assembly quality” from ( Rhie *et al.*, 2021). ** BUSCO scores based on the actinopterygii_odb10 BUSCO set using v5.3.2. C = complete [S = single copy, D = duplicated], F = fragmented, M = missing, n = number of orthologues in comparison. A full set of BUSCO scores is available at https://blobtoolkit.genomehubs.org/view/fPunPun2.1/dataset/CASGFK01/busco.

**Figure 3.**
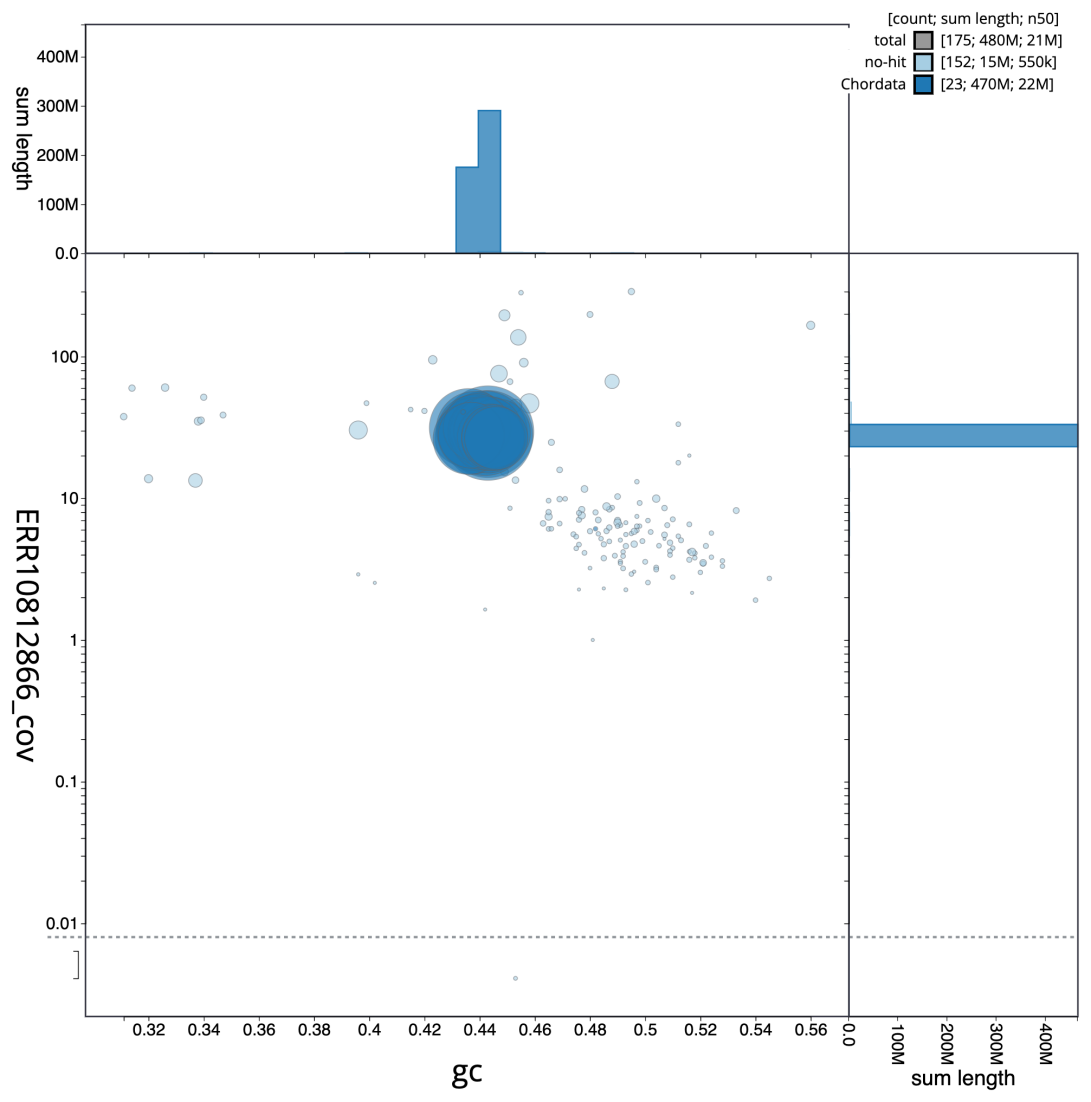
Genome assembly of *Pungitius pungitius*, fPunPun2.1: BlobToolKit GC-coverage plot. Scaffolds are coloured by phylum. Circles are sized in proportion to scaffold length. Histograms show the distribution of scaffold length sum along each axis. An interactive version of this figure is available at https://blobtoolkit.genomehubs.org/view/fPunPun2.1/dataset/CASGFK01/blob.

**Figure 4.**
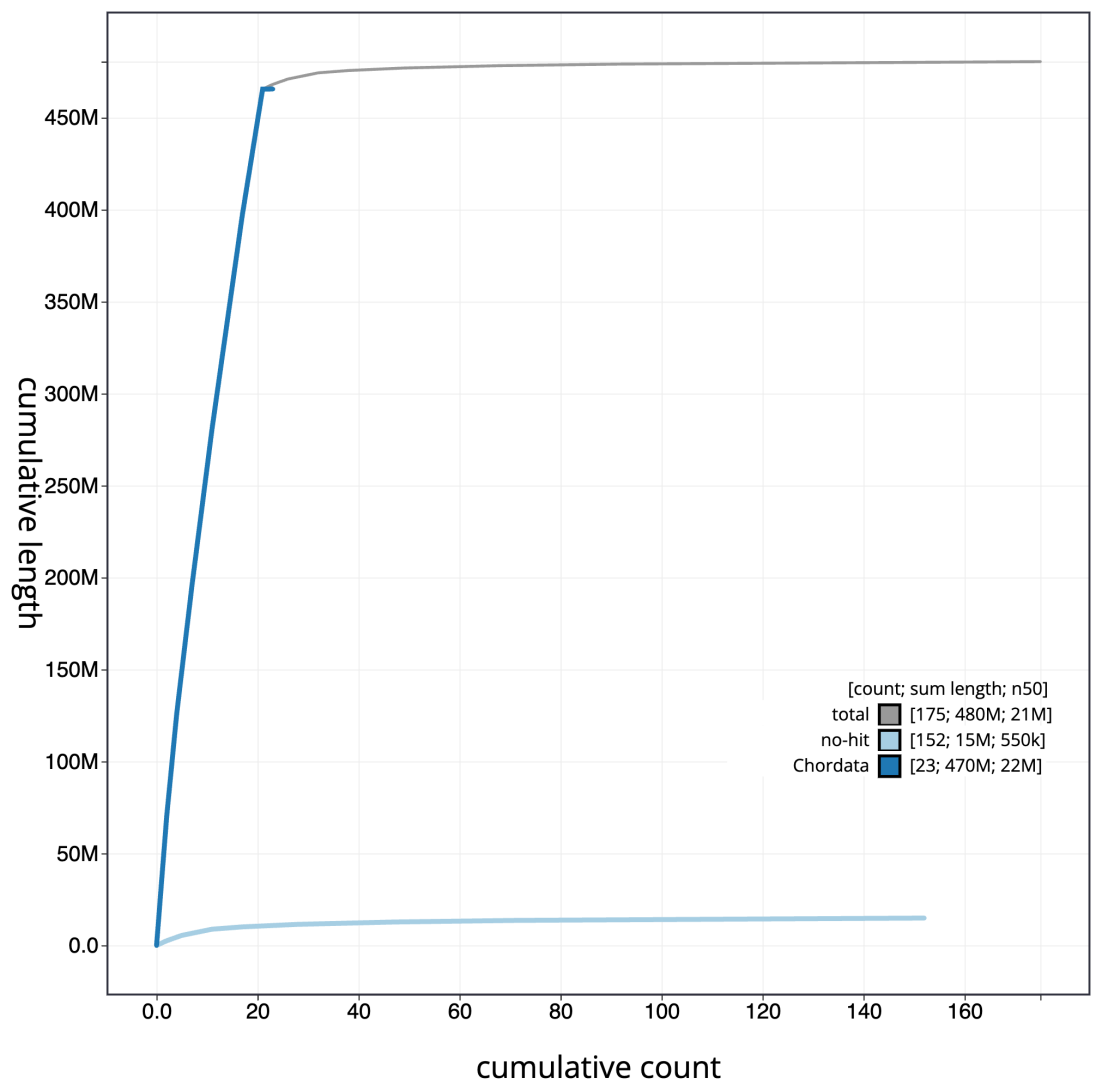
Genome assembly of *Pungitius pungitius*, fPunPun2.1: BlobToolKit cumulative sequence plot. The grey line shows cumulative length for all scaffolds. Coloured lines show cumulative lengths of scaffolds assigned to each phylum using the buscogenes taxrule. An interactive version of this figure is available at https://blobtoolkit.genomehubs.org/view/fPunPun2.1/dataset/CASGFK01/cumulative.

**Figure 5.**
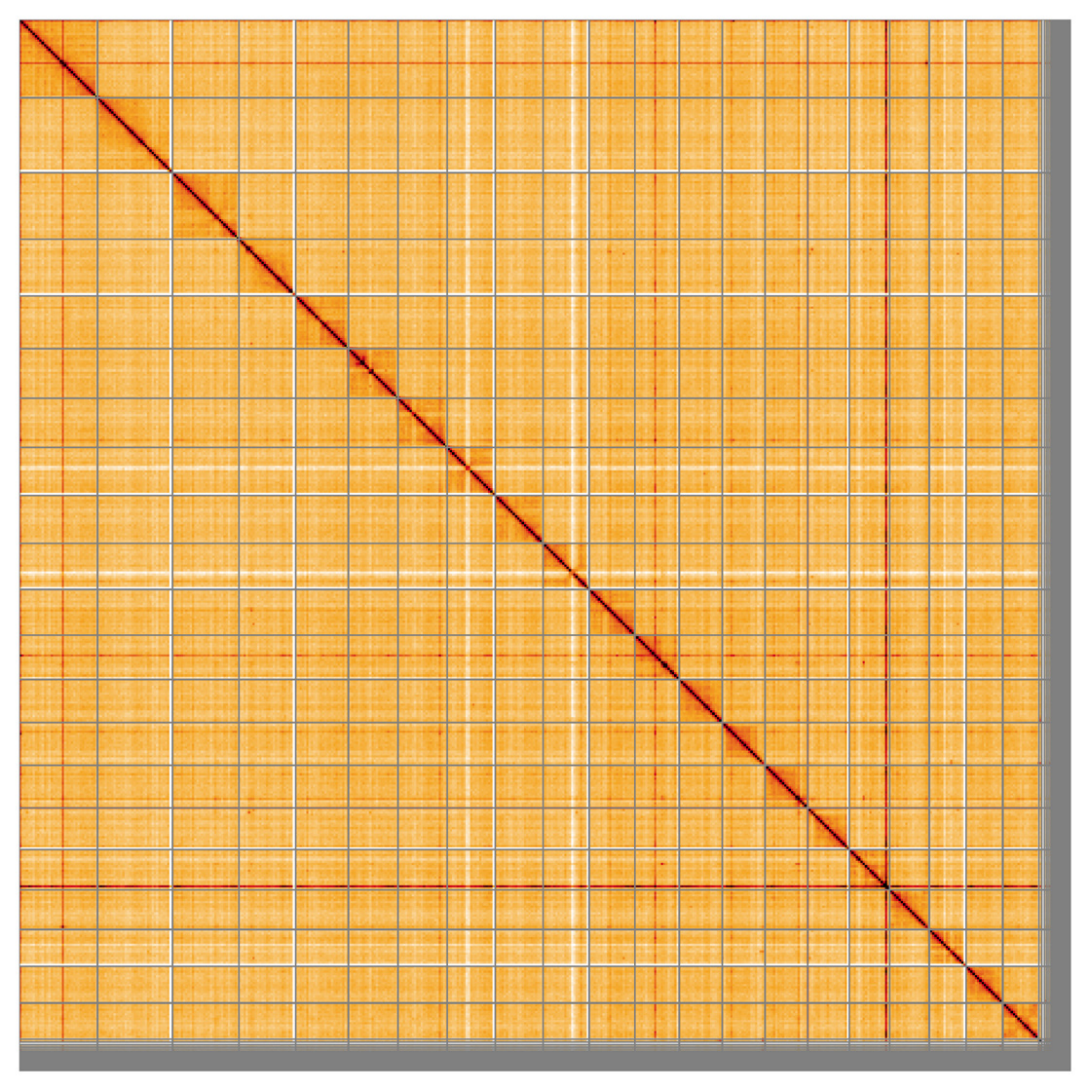
Genome assembly of *Pungitius pungitius*, fPunPun2.1: Hi-C contact map of the fPunPun2.1 assembly, visualised using HiGlass. Chromosomes are shown in order of size from left to right and top to bottom. An interactive version of this figure may be viewed at https://genome-note-higlass.tol.sanger.ac.uk/l/?d=MCjOpVtaT4mHi8fRg1_3Dw.

**Table 2.** Chromosomal pseudomolecules in the genome assembly of *Pungitius pungitius*, fPunPun2.

INSDC accession	Chromosome	Length (Mb)	GC%
OX438541.1	1	35.75	44.5
OX438542.1	2	34.23	44.5
OX438543.1	3	30.3	44.0
OX438544.1	4	25.92	43.5
OX438545.1	5	24.13	44.0
OX438546.1	6	22.6	43.5
OX438548.1	8	22.49	44.0
OX438547.1	7	21.94	43.5
OX438549.1	9	21.81	44.5
OX438550.1	10	21.01	43.5
OX438551.1	11	20.94	44.0
OX438552.1	12	20.22	44.0
OX438553.1	13	19.61	44.0
OX438554.1	14	19.55	44.5
OX438555.1	15	19.44	44.5
OX438556.1	16	18.95	44.0
OX438557.1	17	18.48	44.5
OX438558.1	18	18.07	43.5
OX438559.1	19	16.8	44.5
OX438560.1	20	16.73	44.5
OX438561.1	21	16.56	44.5
OX438562.1	MT	0.02	45.5

The estimated Quality Value (QV) of the final assembly is 55.9 with *k*-mer completeness of 99.99%, and the assembly has a BUSCO v5.3.2 completeness of 97.9% (single = 97.1%, duplicated = 0.7%), using the actinopterygii_odb10 reference set ( *n* = 3,640).

Metadata for specimens, spectral estimates, sequencing runs, contaminants and pre-curation assembly statistics can be found at https://links.tol.sanger.ac.uk/species/134920.

## Methods

### Sample acquisition and nucleic acid extraction

A male *Pungitius pungitius* (specimen ID SAN0000711, individual fPunPun2) was collected from Thwaite Lake, Cottingham, UK (latitude 53.78, longitude –0.40) on 2020-06-25. The specimen was taken from the pond by Bernd Hänfling using a hand net, and identified by Alan Smith. The specimen was transported alive to the University of Hull and left to recover fully in an aquarium for a week before any sampling commenced. The specimen was euthanised in a lethal dose of MS-222 and tissue dissection was carried out by Bernd Hänfling within 30 minutes of euthanasia, and the tissues were immediately shock-frozen in liquid nitrogen.

DNA was extracted at the Tree of Life laboratory, Wellcome Sanger Institute (WSI). The fPunPun2 sample was weighed and dissected on dry ice with tissue set aside for Hi-C sequencing. Muscle tissue was disrupted using a Nippi Powermasher fitted with a BioMasher pestle. High molecular weight (HMW) DNA was extracted using the Qiagen MagAttract HMW DNA extraction kit. HMW DNA was sheared into an average fragment size of 12–20 kb in a Megaruptor 3 system with speed setting 30. Sheared DNA was purified by solid-phase reversible immobilisation using AMPure PB beads with a 1.8X ratio of beads to sample to remove the shorter fragments and concentrate the DNA sample. The concentration of the sheared and purified DNA was assessed using a Nanodrop spectrophotometer and Qubit Fluorometer and Qubit dsDNA High Sensitivity Assay kit. Fragment size distribution was evaluated by running the sample on the FemtoPulse system.

RNA was extracted from muscle tissue of fPunPun2 in the Tree of Life Laboratory at the WSI using TRIzol, according to the manufacturer’s instructions. RNA was then eluted in 50 μl RNAse-free water and its concentration assessed using a Nanodrop spectrophotometer and Qubit Fluorometer using the Qubit RNA Broad-Range (BR) Assay kit. Analysis of the integrity of the RNA was done using Agilent RNA 6000 Pico Kit and Eukaryotic Total RNA assay.

### Sequencing

Pacific Biosciences HiFi circular consensus DNA sequencing libraries were constructed according to the manufacturers’ instructions. Poly(A) RNA-Seq libraries were constructed using the NEB Ultra II RNA Library Prep kit. DNA and RNA sequencing was performed by the Scientific Operations core at the WSI on Pacific Biosciences SEQUEL II (HiFi) and Illumina NovaSeq 6000 (RNA-Seq) instruments. Hi-C data were also generated from muscle tissue of fPunPun2 using the Arima2 kit and sequenced on the Illumina NovaSeq 6000 instrument.

### Genome assembly, curation and evaluation

Assembly was carried out with Hifiasm ( Cheng *et al.*, 2021) and haplotypic duplication was identified and removed with purge_dups ( Guan *et al.*, 2020). The assembly was then scaffolded with Hi-C data ( Rao *et al.*, 2014) using YaHS ( Zhou *et al.*, 2023). The assembly was checked for contamination and corrected as described previously ( Howe *et al.*, 2021). Manual curation was performed using HiGlass ( Kerpedjiev *et al.*, 2018) and Pretext ( Harry, 2022). The mitochondrial genome was assembled using MitoHiFi ( Uliano-Silva *et al.*, 2023), which runs MitoFinder ( Allio *et al.*, 2020) or MITOS ( Bernt *et al.*, 2013) and uses these annotations to select the final mitochondrial contig and to ensure the general quality of the sequence.

A Hi-C map for the final assembly was produced using bwa-mem2 ( Vasimuddin *et al.*, 2019) in the Cooler file format ( Abdennur & Mirny, 2020). To assess the assembly metrics, the *k*-mer completeness and QV consensus quality values were calculated in Merqury ( Rhie *et al.*, 2020). This work was done using Nextflow ( Di Tommaso *et al.*, 2017) DSL2 pipelines “sanger-tol/readmapping” ( Surana *et al.*, 2023a) and “sanger-tol/genomenote” ( Surana *et al.*, 2023b). The genome was analysed within the BlobToolKit environment ( Challis *et al.*, 2020) and BUSCO scores ( Manni *et al.*, 2021; Simão *et al.*, 2015) were calculated.

Table 3 contains a list of relevant software tool versions and sources.

**Table 3.** Software tools: versions and sources.

Software tool	Version	Source
BlobToolKit	4.0.7	https://github.com/blobtoolkit/blobtoolkit
BUSCO	5.3.2	https://gitlab.com/ezlab/busco
Hifiasm	0.16.1-r375	https://github.com/chhylp123/hifiasm
HiGlass	1.11.6	https://github.com/higlass/higlass
Merqury	MerquryFK	https://github.com/thegenemyers/MERQURY.FK
MitoHiFi	2	https://github.com/marcelauliano/MitoHiFi
PretextView	0.2	https://github.com/wtsi-hpag/PretextView
purge_dups	1.2.3	https://github.com/dfguan/purge_dups
sanger-tol/genomenote	v1.0	https://github.com/sanger-tol/genomenote
sanger-tol/readmapping	1.1.0	https://github.com/sanger-tol/readmapping/tree/1.1.0
YaHS	1.2a	https://github.com/c-zhou/yahs

### Wellcome Sanger Institute – Legal and Governance

The materials that have contributed to this genome note have been supplied by a Tree of Life collaborator. The Wellcome Sanger Institute employs a process whereby due diligence is carried out proportionate to the nature of the materials themselves, and the circumstances under which they have been/are to be collected and provided for use. The purpose of this is to address and mitigate any potential legal and/or ethical implications of receipt and use of the materials as part of the research project, and to ensure that in doing so we align with best practice wherever possible. The overarching areas of consideration are:

•   Ethical review of provenance and sourcing of the material

•   Legality of collection, transfer and use (national and international)

Each transfer of samples is undertaken according to a Research Collaboration Agreement or Material Transfer Agreement entered into by the Tree of Life collaborator, Genome Research Limited (operating as the Wellcome Sanger Institute) and in some circumstances other Tree of Life collaborators.

## Data Availability

European Nucleotide Archive: *Pungitius pungitius* (ninespine stickleback). Accession number PRJEB59310; https://identifiers.org/ena.embl/PRJEB59310 ( Wellcome Sanger Institute, 2023). The genome sequence is released openly for reuse. The *Pungitius pungitius* genome sequencing initiative is part of the Darwin Tree of Life (DToL) project. All raw sequence data and the assembly have been deposited in INSDC databases. The genome will be annotated using available RNA-Seq data and presented through the Ensembl pipeline at the European Bioinformatics Institute. Raw data and assembly accession identifiers are reported in Table 1.

## Author information

Members of the Wellcome Sanger Institute Tree of Life programme are listed here: https://doi.org/10.5281/zenodo.4783585.

Members of Wellcome Sanger Institute Scientific Operations: DNA Pipelines collective are listed here: https://doi.org/10.5281/zenodo.4790455.

Members of the Tree of Life Core Informatics collective are listed here: https://doi.org/10.5281/zenodo.5013541.

Members of the Darwin Tree of Life Consortium are listed here: https://doi.org/10.5281/zenodo.4783558.
