# Supplementary material for: The genome sequence of the nine-spined stickleback, Pungitius pungitius (Linnaeus, 1758)
Source: Wellcome Open Res. 2023 Dec 5;8:555. [Version 1] doi: 10.12688/wellcomeopenres.20354.1 (PMC11303943; doi:10.12688/wellcomeopenres.20354.1)
Supplement: Peer review history

# Reviewer report: Reviewer response for version 1

**DOI:** 10.21956/wellcomeopenres.22539.r88519

**Published:** 2024-08-06

**Authors:** Frederic Chain, Michael Olufemi

**Affiliations:** 1 Biological Sciences, University of Massachusetts Lowell, Lowell, Massachusetts, USA

The nine-spined stickleback, *Pungitius pungitius*, is an important ecological and evolutionary study organism alongside other stickleback species. Its genome provides a resource for understanding the genetic basis of a wide-range of repeatedly evolved phenotypes across populations and species. The protocols, methods, and datasets are overall clearly presented.

**Comments:**

- I wonder to what extent this assembly differs from others. The study by Varadharajan *et al.* is mentioned in the Background, but it isn't clear how different the assemblies are (or the origin of the individual sample used for sequencing). There have also been more recent improvements to this other reference Kivikoski M *et al. (*2021 ^1^ ) and Wang D *et al. (*2024 ^2^ ), which include attempts to identify and separate the X and Y chromosomes. The most recent assembly appears to have superior assembly metrics and BUSCO completeness. A comprehensive comparative analysis is not necessary but it seems relevant to at least reference these articles.
- **Manual Curation:** Please provide definitions and separate numbers for missing joins and mis-joins for clarity.
- **Metadata:** I could not access the data link https://links.tol.sanger.ac.uk/species/134920
- **Sex Chromosome Identification:** The data note lacks an explanation for the inability to identify the sex chromosome despite sequencing the heterogametic sex. The authors may also refer to the recently published article that identifies the Y chromosome in another assembly: Wang D *et al. (*2024 ^2^ )
- **Figure 3:** More information would be helpful here for the naive reader on how these data are generated. What does 'scaffolds are colored by phylum' mean and what does 'no-hit' represent? This is explained a bit in Figure 4 - that scaffolds are assigned by busco genes tax rules, but it isn't explicitly mentioned in the main text.
- **Genome annotation:** I was hoping for annotation data alongside the genome data. The RNA-seq data is mentioned several times but only at the end of the article do we find out that it will be used in the future for genome annotations - I recommend mentioning this earlier in the data note.

Are sufficient details of methods and materials provided to allow replication by others?

Partly

Is the rationale for creating the dataset(s) clearly described?

Yes

Are the datasets clearly presented in a useable and accessible format?

Yes

Are the protocols appropriate and is the work technically sound?

Yes

Reviewer Expertise:

Evolutionary Genomics and Bioinformatics

We confirm that we have read this submission and believe that we have an appropriate level of expertise to confirm that it is of an acceptable scientific standard.

## References

[1] : Automated improvement of stickleback reference genome assemblies with Lep-Anchor software. *Mol Ecol Resour* .2021;21(6) : 10.1111/1755-0998.13404 2166-2176 10.1111/1755-0998.13404 33955177

[2] : Improved assembly of the Pungitius pungitius reference genome. *G3 (Bethesda)* .2024; 10.1093/g3journal/jkae126 10.1093/g3journal/jkae126 38861393 PMC11304971

# Reviewer report: Reviewer response for version 1

**DOI:** 10.21956/wellcomeopenres.22539.r88518

**Published:** 2024-07-23

**Authors:** Heiner Kuhl

**Affiliations:** 1 Leibniz-Institute of Freshwater Ecology and Inland Fisheries, Berlin, Germany

Hänfling et al. present a genome assembly of nine-spined stickleback ( *Pungitius pungitius*). The sequencing technologies used are sound (HIFI + Hi-C). 

**Minor:**

 There is a *Pungitus pungitus* genome from 2020 (GCF_902500615.1). The introduction mentions such prior work, but some comparisons should be done in the manuscript to show potential advantages of the new assembly.

 Some of the assembly statistics (contig N50 = 1.4Mbp) seem to be much lower than expected for HIFI reads, which can yield T2T assemblies of some chromosomes in fish species with much larger and more repetitive genomes. It should be discussed, what was limiting the assembly continuity (contig-level). In this regard HIFI readlength distribution, estimates of heterozygosity and a repeat analysis should be presented.

 The authors state: "While not fully phased, the assembly deposited is of one haplotype." It seems to be a standard phrase in this type of articles and as mentioned before I am not sure if this is correct. If I read the methods correctly, the chromosomal scaffolds will represent a mosaic of the two haplotypes. Or has Hi-C data been used to disentangle chromosomal haplotypes during hifiasm? This needs to be clarified.

 The authors state "Sheared DNA was purified by solid-phase reversible immobilisation using AMPure PB beads with a 1.8X ratio of beads to sample to remove the shorter fragments and concentrate the DNA sample", this also seems to be a standard phrase in articles of this type. But looking at the PacBio protocols it seems the ratio needs to be much higher (at least 3.1X) to not loose your DNA ( Procedure & Checklist – Using AMPure PB Beads for Size-Selection (pacb.com)).

Are sufficient details of methods and materials provided to allow replication by others?

Yes

Is the rationale for creating the dataset(s) clearly described?

Yes

Are the datasets clearly presented in a useable and accessible format?

Yes

Are the protocols appropriate and is the work technically sound?

Partly

Reviewer Expertise:

Genomics, Bioinformatics, DNA/RNA Sequencing technologies

I confirm that I have read this submission and believe that I have an appropriate level of expertise to confirm that it is of an acceptable scientific standard, however I have significant reservations, as outlined above.

# Reviewer report: Reviewer response for version 1

**DOI:** 10.21956/wellcomeopenres.22539.r82406

**Published:** 2024-05-25

**Authors:** Fernando Cruz

**Affiliations:** 1 Genome Assembly and Annotation Team, Centro Nacional de Análisis Genómico (CNAG), C/Baldiri Reixac 4, 08028 Barcelona, Spain. Universitat de Barcelona (UB), Barcelona, Spain, Barcelona, Spain

**Summary of the article**

 The genome sequence presented here corresponds to the most diverse and evolutionary successful genus in the Family *Gasterosteidae*. The methods and approaches followed ensure the public release of a chromosome-level assembly far above the minimum assembly standards required by the Vertebrate Genomes Project. Finally, the nine-spined stickleback is a “supermodel” organism to study multiple biological questions such as the phenotypic plasticity. (see reference Juha Merilä, 2013)

**Main comments and suggestions**

 1. Introduction: you state state “nine congeneric species, compared to the three for *G. aculeatus*, ” However the GenBank Taxonomy shows at least 5 species for Gasterosteus genus: G.aculeatus, G.gymnurus, G.islandicus, G. niponicus and G. wheatlandi. Please revise if the statement is correct.

 2. Manual curation paragraph in page 3: the authors pool the numbers of missing joins and mis-joins into one category. Could authors give the number for each of them? *Manual assembly curation corrected **44 missing joins or mis-joins** and removed 3 haplotypic duplications* This could be confusing for some readers that are not very familiar with genome assembly. For example, thinking that missing joins (pieces that were not joined by the scaffolder but should be join based on their contacts) are the same as a misjoin (a missassembly).

 3. Metadata: Please revise the link provided for the Metadata ( https://links.tol.sanger.ac.uk/species/134920) it yields this error with 3 different browsers (Safari, Chrome and Firefox): “ *This XML file does not appear to have any style information associated with it. The document tree is shown below.”*

 3. Heterogametic Sex: Could you explicitly say that this species has an XY determination system? It will be good for the non-specialists in the organism. I personally think is good practice to specify that the sequenced specimen corresponds to the heterogametic sex. https://www.ncbi.nlm.nih.gov/pmc/articles/PMC6329827/

 4. Sex Chromosome Identification: The species is supposed to have heteromorphic sex chromosomes ( https://doi.org/10.1111/j.1095-8649.2008.01902.x). Could you provide a succinct explanation or reason why you could not tag the sex chromosome?

**Minor suggestions**

 In the references, it would be better if the link points to the species IUCN assessment, Perhaps, adding a more specific title The IUCN Red List of Threatened Species: *Pungitius pungitius* IUCN Assessment (2013) https://www.iucnredlist.org/species/18878/18236204

Are sufficient details of methods and materials provided to allow replication by others?

Partly

Is the rationale for creating the dataset(s) clearly described?

Yes

Are the datasets clearly presented in a useable and accessible format?

Yes

Are the protocols appropriate and is the work technically sound?

Yes

Reviewer Expertise:

Bioinformatics, Genomics, Evolutionary Biology and Biodiversity

I confirm that I have read this submission and believe that I have an appropriate level of expertise to confirm that it is of an acceptable scientific standard.

## References

[1] : The evolution of sex determination associated with a chromosomal inversion. *Nat Commun* .2019;10(1) : 10.1038/s41467-018-08014-y 145 10.1038/s41467-018-08014-y 30635564 PMC6329827

[2] : Heteromorphic sex chromosomes in the ninespine sticklebackPungitius pungitius. *Journal of Fish Biology* .2008;73(2) : 10.1111/j.1095-8649.2008.01902.x 456-462 10.1111/j.1095-8649.2008.01902.x
